# Our Sham Civilisation

**Published:** 1902-07-05

**Authors:** 


### Editor's Letter-Box.

[Our Correspondents are reminded that prolixity is a great bar to publication, and that brevity of style and conciseness of statement greatly facilitate early insertion.]

### "OUR SHAM CIVILISATION."

We are informed that the insanitary restaurant to which we alluded in an article with the above heading in our issue of June 21 is not "in" the Strand, and further that it is not within the boundaries of the City of Westminster. We, however, never stated that it was either one or the other, having merely described the place as it was described at the meeting of the Epidemiological Society, as being "near the Strand" which does not seem to be disputed. However, our correspondent agrees with our strictures as to its condition, and adds that he has heard that the house in question was to be pulled down as soon as the patient referred to could be removed.

